# Universal health coverage evolution, ongoing trend, and future challenge: A conceptual and historical policy review

**DOI:** 10.3389/fpubh.2023.1041459

**Published:** 2023-02-03

**Authors:** Chhabi Lal Ranabhat, Shambhu Prasad Acharya, Chiranjivi Adhikari, Chun-Bae Kim

**Affiliations:** ^1^Department of Health Promotion and Administration, College of Health Science, Eastern Kentucky University, Richmond, KY, United States; ^2^Global Center for Research and Development, Kathmandu, Nepal; ^3^Country Strategy and Support, World Health Organization, Geneva, Switzerland; ^4^School of Health and Allied Science, Pokhara University, Pokhara, Nepal; ^5^Indian Public Health-Gandhinagar, Gujarat, India; ^6^Department of Preventive Medicine, Wonju College of Medicine, Yonsei University, Wonju, Republic of Korea

**Keywords:** universal health coverage (UHC), health financing, conceptual analysis, social health protection, health service access, population and financial coverage, historical and policy review

## Abstract

The goal of universal health coverage (UHC) from the United Nations (UN) has metamorphized from its early phase of primary health care (PHC) to the recent sustainable development goal (SDG). In this context, we aimed to document theoretical and philosophical efforts, historical analysis, financial and political aspects in various eras, and an assessment of coverage during those eras in relation to UHC in a global scenario. Searching with broad keywords circumadjacent to UHC with scope and inter-disciplinary linkages in conceptual analysis, we further narrated the review with the historical development of UHC in different time periods. We proposed, chronologically, these frames as eras of PHC, the millennium development goal (MDG), and the ongoing sustainable development goal (SDG). Literature showed that modern healthcare access and coverage were in extension stages during the PHC era flagshipped with “health for all (HFA)”, prolifically achieving vaccination, communicable disease control, and the use of modern contraceptive methods. Following the PHC era, the MDG era markedly reduced maternal, neonatal, and child mortalities mainly in developing countries. Importantly, UHC has shifted its philosophic stand of HFA to a strategic health insurance and its extension. After 2015, the concept of SDG has evolved. The strategy was further reframed as service and financial assurance. Strategies for further resource allocation, integration of health service with social health protection, human resources for health, strategic community participation, and the challenges of financial securities in some global public health concerns like the public health emergency and travelers' and migrants' health are further discussed. Some policy departures such as global partnership, research collaboration, and experience sharing are broadly discussed for recommendation.

## Background

Universal health coverage (UHC) means that the whole universe's population has access to all types of healthcare. It refers to a government system or program that guarantees that all people under that government have access to available health services. The system will provide such services when and as required without causing financial challenges for the individual receiving such services. UHC programs by design offer all essential and quality health services, namely, health promotion, preventive health, medical treatment, rehabilitation, palliative care, and hospice care (1).

Despite the core definition, UHC is fundamentally a human right and political scheme. The human rights-based approach (HRBA) has established its legacy in inclusive development that follows the United Nations Development Program (UNDP)'s human development approach and integrates standards and principles of human rights, such as participation, non-discrimination, and accountability (2). It provides a procedural way for implementing UHC at the national level and concludes by highlighting critical areas in which consistent, authoritative, and practical guidance is urgently needed to support countries in getting onto the right road to UHC (3, 4). In the same line, addressing UHC requires changing a wide spectrum of laws, policies, and practices that reflect the willingness and capacity of governments to deliver on their commitments and meet their human rights obligations. UHC has been established in a wider, longer, and deeper journey toward the realization of human rights using various legal, historical, institutional, and social arguments.

Globally, there is no consistency in providing healthcare in terms of access, equity, and quality. The UHC concept was developed during the millennium development goal (MDG), 2000–2015. There was a challenge in developing the healthcare package to achieve UHC in Malawi (5). There is some confusion when setting the priority and designing the policy in Uganda (6) due to the conceptual unclarity of UHC. Previous assessments have mainly focused on the provision of essential services, the availability of healthcare resources, and health service utilization rates in high-income countries rather than in low-income and middle-income countries (7). A study in China has revealed that there is some unclarity about the resource pooling in healthcare for ongoing health insurance programs (8). There is a power imbalance in the global governance sectors, health disparities, few choices in health service access, and institutional barriers according to *The Lancet-University of Oslo Commission Report* (9). Due to the different dimensions of the power structure, there has been a shift in obligation from public provision of health services to an individualized responsibility for health outcomes where health is increasingly commodified and citizens are recast as consumers (10). In low- and middle-income countries, political destination, governance, and resource allocation are lacking to achieve UHC (11). On the other hand, proper health financing modality is a pathway to achieving UHC. Inappropriate health financing models and disproportional resource allocation are bottlenecks that can hinder achieving UHC (11). Since UHC is a multisectoral and multidimensional issue with an ambitious health goal, careful and smart resource distribution in healthcare is needed. There is also a need for conceptual and contextual clarity in UHC. There is no uniformity in the conceptual definition or scope of UHC, including whether UHC is achievable, how to move forward, common indicators for measuring its progress, regular monitoring of those indicators, and clear interpretations of those indicators (12). Therefore, conceptual clarity, proper measurement, and the formation of community-based essential healthcare package are needed (13). An American education publication company has suggested that a link of historical development of healthcare with UHC should be mandatory to track those indicators (14). The aim of this study was to describe theoretical and philosophical efforts, historical analysis, financial and political aspects in various eras, and an assessment of coverage during those eras in relation to UHC. A review of past efforts, analysis of current trends, and clear future directions are necessary to achieve UHC.

## Methodology

We used a general review approach to dissect UHC from multiple perspectives. To be more focused, conceptual and historical developments toward UHC have been presented from the available literature. We fixed the article's content in light of policy and program confusions in various countries (15), the multiple foundations of UHC (16), gaps in healthcare equity and excess during historical paradigm shifts (17), and the current and future challenges on the path of UHC (18). The future challenges for UHC presented in this article were articulated on the basis of epidemiological, demographic, existing policy discrepancies, and lifestyle factors associated with healthcare (11, 19). We selected the literature in 4 steps as follows: (1) search the literature by keywords through Google, (2) sort the title by those that best match in first 5 webpages, (3) go for full access to the literature from the titles, and (4) pick up it, if the paper is useful or discard it. Major keywords searched for studies were universal health coverage, health disparity, healthcare access, equity, philosophy, historical development, conceptual analysis, social health protection, public health emergency, and so on. The nature of our study does not demand hard inclusion and exclusion criteria. Regarding the quality of literature, almost all were taken from peer-reviewed journals, books, and reports, and all references are available online. A concept analysis is a process to guide the explanation of a concept that might be vague, ambiguous, confusing, or incomplete (20). It is a well-established methodology in public health practice that is used to examine many contents, which are key for preventive health and health promotion including cultural aspect (21), empowerment (22), participation (23), equity (24), and health literacy (25). This study adopted an evolutionary concept analysis process developed by Rodgers (26) to incorporate ideas by Risjord (26, 27). This concept analysis needs to explore contextuality in terms of time, place, and discipline. In contrast, a “theoretical concept analysis” aims to represent the concept as it appears in a particular body of scientific and theoretical literature. A concept analysis is often used to explore new and underdeveloped concepts and theories. Additionally, it can also be used to clarify and define concepts that are open to individual interpretation, multiple truths, and subjectivity (28).

Historical analysis was performed for health service equity, access, and quality in different stages as a method where the gradual development of UHC was ovulated. We presented healthcare philosophy, theories, and policy practices in three historical periods, namely, health for all (1978–2000), millennium development goal (2000–2015), and ongoing sustainable development goal (SDG, 2015–2030), which includes WHO's 13th General Program of Work (WHO GPW13) period, 2018–2025 (with extension agreed by its member states). We presented health service coverage, financial service coverage, and population coverage using historical trend analysis of modern healthcare. Furthermore, issues of healthcare equity and access have been linked to politics, health financing, human right, and each individual responsibility (Table 1).

**Table 1:** Review approach, conceptual, and historical analysis summary.

**Searching keywords**	**Conceptual analysis**	**Historical review**
Universal health coverage, health, healthcare access, equity, philosophy, historical development, conceptual analysis, social health protection, public health emergency etc.	Terminological analysis, Philosophical and Theoretical analysis, Political analysis, Linkage with health financing, Linkage with Social health protection (SHP)	• Before primary health care (PHC) stage • PHC phase • Millennium Development Goal phase • Sustainable development goal phase

## Results

We performed a conceptual analysis for terminological clarity, theory, philosophy of UHC, and its core linkage with politics, health financing, and social health protection. A historical review was performed to understand how modern healthcare started and chronologically developed globally. Historical phases are divided into before PHC (before 1978), PHC era (1978–2000), millennium development goal (MDG) (2000–2015), and SDG (2015–2030). In these historical periods, there are health priorities, major achievements, and measuring indicators.

### Terminological clarity

From an etymological perspective, *Universal* means “everyone”. It is similar to the Universal Declaration of Human Right and *Health* as defined by the WHO constitution (29). *Coverage* resonates with protection, as does the fundamental human rights principle. Similarly, coverage is linked to social protection under SDG 1.3, which in turn can fasten human right to social security (30). The Committee on Economic, Social, and Cultural Rights has interpreted coverage as all people covered by the social security system, especially individuals belonging to the most disadvantaged and marginalized groups without discrimination. It has been noted that schemes are necessary to ensure “universal coverage” but not necessarily mandatory contributions (31).

### Theoretical and philosophical ground of universal health coverage (UHC)

UHC implicates a wide array of human rights, including rights to life, health, security, equality and non-discrimination, the standard of living, freedom of movement, association, assembly, information, expression of thought, social security, privacy, participation, a basic standard of living like water, food, housing, education, and access to benefits of scientific progress. These and other rights are protected in international and regional treaties and national constitutions. They also form part of customary international law. Overall, they can be traced back to the Universal Declaration of Human Rights, which has established the groundwork for the international human rights movement (29).

The Universal Declaration of Human Rights was derived from discrimination and inequality of global polarization during the Second World War shortly after the establishment of the UN, with human rights as one of its foundational purposes (32). In this spirit, the WHO Constitution (1946) sets the standard for the highest attainable standard of health as a human right (33). During this period, many industrialized countries emerging from the devastation of the war established their health systems (e.g., France in 1945, the United Kingdom in 1948, and Japan in 1951). To this date, these systems are integral to the wider governance of society, as reflected in the Alma-Ata and Astana declarations on PHC, which reaffirm governments' responsibility to promote the health of their people. Health is referred to as the foundation of human rights (34). Furthermore, specific legislation and jurisprudence demonstrate how human rights norms and principles should allow national health systems and set parameters for what governments as stewards of these systems can and should do as well as what they are restricted to do (35). The General Assembly resolutions of WHO to adopt UHC over the years have consistently advocated how human rights—particularly the right to health—provide the overarching framework for UHC (36). In a similar vein, the UN special rapporteur on the right to health has emphasized that UHC must be understood as a right to health (37).

### Political aspect

UHC is fundamentally a political agenda. In the world of global health governance, UHC is part of an ongoing debate about the relative importance of vertical priorities, individual wellbeing, disease control, eradication, and broader horizontal, health system-strengthening proposals. It is not possible without political priority because there is a need of willpower for health system strengthening and resource allocation for wellbeing, disease control, and prevention (38, 39).

The effects of widespread democratization from the 1970's to the late 1990's can help us explain the expansion of UHC in middle-income countries today. Different authoritarian regimes had less accountability to the broad population with a poor commitment toward organized challenges. Those authoritarians were dictatorship in nature, state resources were mobilized to their families, and health services were available to a limited number of people as directed by those authoritarians (40–42). One of the political systems is the cadre-based political system, and it is one of the most promising paths to explore UHC. In particular, left-wing parties are more likely to enact redistributive policies in the spirit of UHC (43, 44). Socialist parties enacted universal healthcare across southern Europe when they came to power after democratization despite major recessions that might block health access expansion. Later, it was the left that universalized healthcare in Latin American countries such as Brazil and Chile (45, 46). At times, conservative parties have also expanded health coverage for their own political purposes at times (47). Otto von Bismarck created the first social health insurance in response to socialist challenges. Japan's health insurance expansion was the result of the left labor challenge to the dominant conservative Liberal Democratic Party. The expansion of healthcare access in Mexico was partly a strategy to maintain the popularity of its once-dominant Party of the Institutional Revolution (48, 49). It shows that appropriate political vision, conviction, commitment, and related policies engineering are decisive factors to achieve UHC. However, the political nature, ideology, and governance are equally important for healthcare equity, access acceptability, and quality.

### UHC and healthcare financing

The concept of healthcare financing schemes is an application and extension of the concept of social protection schemes (50). Health financing systems mobilize and allocate budget within the health system to meet the current health needs of the population (individual and collective) with an anticipated view of futuristic needs. There should be more choices of healthcare providers and payment modalities (such as direct payment through a third party) and mechanisms developed by the state (such as volunteer insurance, national health service, and social insurance) (51). Normally, there are four types of healthcare models. Each model is distinct in and of itself. Most countries do not strictly adhere to a single model. Rather, they create a hybrid model that fits their context.

### The Beveridge model

Developed in 1948 by Sir William Beveridge in the United Kingdom, the Beveridge model is often centralized through the establishment of a national health service (52). Fundamentally, there is a single-payer government system with a low cost and a standard benefit. The service is available on their networks. It is fully funded by taxes without needing out-of-pocket payments or cost sharing. Every tax-paying citizen is guaranteed the same access to care. Nobody will ever receive a medical bill. There is a potential risk of overutilization of the Beveridge Model (53). Due to a high demand of healthcare with free access, there is a chance of rising costs and demands for higher taxes. Thus, many of these systems have regulations in place to manage healthcare demands.

### The Bismarck model

The Bismarck model is very popular. It was created near the end of the 19th century by Otto von Bismarck as a more decentralized form of healthcare. In this model, employers and employees are both responsible for paying the health insurance premium through sickness funds created by payroll deductions (54). Regardless of a preexisting condition, private companies cover all types of services for each employee. Those plans are not meant to be profitable. Normally, healthcare providers are private, whereas insurers are public. In some instances, there is a single insurer (France, Korea). However, in other countries, like Germany and the Czech Republic, there are multiple competing insurers. The government controls the price. However, for UHC, each individual needs to contribute in different modalities. The challenge of the Bismarck model is how to sustain healthcare for a vulnerable and aging population. Now, this model has been adopted by Germany, Belgium, Japan, Switzerland, the Netherlands, France, and partially the USA.

### The national health insurance model

The national health insurance model combines different aspects of both the Beveridge model and the Bismarck model. The government acts as the single payer for medical procedures as in the Beveridge model. Similarly, providers are private as in the Bismarck model. This model is driven by private providers, although payments come from a government-run insurance program that every citizen can afford (55). Fundamentally, the national health insurance model is a universal insurance that could not make or concern for a profit or deny claims either. Usually, there is no need for marketing. Moreover, it is cheaper and much simpler to navigate. This balance between private and public gives hospitals and providers more freedom without needing many complexities of insurance plans and policies (56). The national health insurance model has been adopted by US Medicare and the health systems in Taiwan, Canada, and South Korea. The demerit of the national health insurance model is the potential for a long waiting list and delay in treatment, which needs special policy departure to overcome those problems, mainly to become flexible on alternate strategies and not stick to one-size-fits-all (57).

### The out-of-pocket (OOP) model

The OOP model is the most common model in less-developed areas and countries where there are not enough financial resources to create a medical system like the three models above. Patients must pay for their procedures from their pockets. It is like a commodity purchase where wealthy people can afford high-quality and professional medical care while poor people might get state or welfare organization that offers basic health services. Thus, healthcare is still driven by income (58). This model is mainly adopted by India, most Asian and African countries, South America, and uninsured or underinsured populations in the United States.

UHC has a set of objectives that health systems pursue. It is not a simple scheme or a particular set of arrangements in the health system. Making progress toward UHC is not limited to increasing the percentage of the population in an explicit insurance scheme. In countries like Germany and Japan, insurance schemes are used to ensure financial access and financial protection for all populations. In countries such as Sweden, the United Kingdom, and Northern Ireland, financial access and financial protection for all are achieved without anything called an insurance scheme. In most low- and middle-income countries (LMIC), free services are somehow legalized and promised. However, they are far from poor people access victimized by catastrophic health expenditures. In summary, it can be concluded that health financing models are just tools for health equity, access, and financial protection. Countries can assemble health financing models according to their contexts.

### Social health protection (SHP) and UHC

Social protection can help individuals and families support their basic needs such as food, housing, and healthcare for vulnerable people (such as the poor, aging, disabled, children, women in difficult conditions, and jobless people) to conduct regular life and promote productivity. It has a series of public or publicly organized and mandated private measures against social distress and economic loss caused by reduction of productivity, stoppage or reduction of earnings, or cost of necessary treatment that can result from ill health (59). The International Labor Organization (ILO) was promoted by the SHP for international development, whereas the UHC for global health by WHO, both two wings of the United Nations (UN). SHP is a special and adequate package for improving health and ultimately enhancing economic growth. A healthier population can create surplus values in work and profession because they are more creative, hardworking, and low cost of disease burden. Well-managed SHP can deliver universal health coverage (UHC) to appropriate healthcare that is accessible and affordable (60). The reestablishment of the world economic structure, which led to accelerated real GDP growth across many low- and middle-income countries (LMICs) (61), might have demanded different and larger benefit packages of social securities all over the world, mainly the LMICs. Basically, those packages are in the form of cash. They can promote human resource productivity. UHC is a consistent healthcare package with the aim of achieving universal coverage in terms of service, population, and complete financial protection. As a result, the programs, resources, and delivery systems have been distributed throughout, increasing the probability of duplication among them. Furthermore, lacking a health technology assessment (HTA) body further jeopardizes the situation in achieving the UHC (62). To prevent duplication of the program, the efficiency of resource integration of SHP and UHC is useful.

## Development and chronology of UHC

### Situation and context of healthcare before PHC: A period of establishing a scientific foundation in healthcare

Before the UN establishment, equal access to healthcare for all people was an out-of-the-box agenda because there was no widely acceptable healthcare service with almost feudal type of governments all over the world (63). The primary features of feudalism were absolutist monarchism, centralization, and hierarchical land ownership. In this system, healthcare was available to all. In addition, there would be bosses rather than leaders. The modern healthcare system was for limited people who had power and resources. The ruling absolute authoritarian persons like popes, including other religious leaders and kings, neither had an agenda to provide accessible health services to all people nor did they have belief in science, research, medicine, or diagnostic tools (64). Rulers set priority on war and romanticism rather than the welfare of the public and the issue of health as a right associated with the welfare of a country.

Before 1950, there was a slow extension of modern health services. There was no uniformity in health services. Most people used ethnomedicines, complementary services, and alternative services. The occurrence of disease and illness in early explanations was based on myths, stories, religious interpretations, and mischief or vengeance. There was an equal practice of Chinese medicine, Japanese practice, and Ayurvedic practice in Asia (65). Due to diversity, belief, and efficacy, a continuous research extension of modern health service was not a priority. If there is no uniform health service and system, health access to all people would be low in priority.

During ancient and medieval periods, health and disease were mainly curative focused and mostly resolved with Spells, Chants, Herbals, Ayurveda, and other Traditional Medicines including Yin-Yang that originated in ancient China. To a meager, some thrusts for participation and health financing from the state level were observed during Lichchhavi (ancient) and Malla (medieval) kingships in Nepal, like state-funded Ayurvedic Health House (Arogyashala), regulating the umbilical cord from the state level, and services to provide without discrimination of caste and ethnicity (66), herbal medicine use in China (67), use of herbal cosmetics in ancient India (68), and such. These systems are rooted in communities; they are easy to use and access, and they even have less frequent and less severe side effects, implicating wider acceptability.

### Primary health care era (1978–2000): Period focusing on community participation in healthcare

In an international conference convened by the World Health Organization (WHO) and United Nations Children's Fund (UNICEF) in Kazakhstan, 134 member countries of WHO ratified the Declaration of Alma-Ata in 1978 (34). The declaration committed member states to support the PHC as a policy to achieve the WHO definition of health (63). PHC was set up for social justice and was created for social reform in Europe and the rapid decolonization of Asia and Africa after the end of the Second World War.

PHC was built on principles of equity in access to health services and the right of people to participate in decisions about their own healthcare (64). Underpinning these principles could deliver preventive and promotive health services, appropriate technology, and intersectoral collaboration (69). It has been argued that PHC begins a shift in health paradigms—from a definition of health limited to biomedical research, the provision of health services by professionals, and institutional care in hospitals and sub-health units such as health centers to a broader focus that includes social determinants of health (70). It is well-known that PHC is a visionary concept that pushes conventional understanding of how health improves from the realm of biomedicine into a realm of social, economic, and political investigation and action.

In 2019, the WHO published a book entitled “Review of 40 Years of Primary Health Care Implementation at Country Level.” It concluded that PHC was the main foundation of UHC and SDG. Political will and governance, global movement of health reform, strengthening the health system for healthcare access and equity, partnership, organization, and management are the main enabling factors. Human resources for health, limited financial resources, inadequate policy frameworks, poor quality of health services, and a health information system are key challenges. Context-specific challenges related to health inequities and access barriers are equally sensitive. More importantly, financial protection of health through health insurance was just started. It was not an issue associated with a program or policy.

Community participation (CP) was proposed as one of the fundamental principles of PHC in the declaration. Community participation and engagement (CPE) gained momentum in this era. Various literature have explored five progressive involvement of the community people and ownership in health programs for the UHC–informing, consulting, collaborating, co-creating/empowering, and horizontal engagement (71, 72). Nepal's female community health volunteer (FCHV) program, as an example of a community-based approach, was initiated in 1988 for family planning purposes, which proved successful and became popular in many other programs child, maternal, and disease control programs, within a couple of years, and was established as a backbone of the healthcare system (73).

### Assessment of coverage

Primary health care was a beginning step in UHC in terms of equity, access, and quality at this time. Health service was measured by immunization against six killer diseases, case findings, and treatment completion of major diseases (TB, leprosy, malaria, and HIV/AIDS) and birth control. Population coverage was < 50%. There was no concept of financial coverage in this phase. Despite the efforts and similar approaches in other countries like Sri Lanka and some African countries, the CPE reached only to a collaborative (3rd) level (74) and was even more critical in the countries going through civil conflicts and war-torn societies (75).

### Millennium development goal and health goal (2000–2015): A period of achieving basic healthcare

Millennium development goals (MDGs) were established following the millennium summit of the United Nations in 2000, after the adoption of the United Nations Millennium Declaration. Each goal had specific targets and a fixed timeline to achieve those targets. A total of eight goals were measured by 21 targets.

In the past 13 years, the MDGs managed to focus world attention and global political consensus on the needs of the poorest to achieve a significant change in Official Development Assistance (ODA) commitments (24). They have provided a framework allowing countries to plan their social and economic development and donors to provide effective support at national and international levels (72). Programs and activities targeted MDGs 4, 5, and 6 in developing countries, focusing on maternal and child health (MCH) and communicable diseases. It has been further criticized that MDGs 4 and 5 were the most important in the African region, while MDGs 7 and 8 were the most important in the Western Pacific region, rather than global perspectives (73). Low-income countries have attached higher relevance to MDG1 than high-income countries (73). Arab countries have not considered the MDGs as a top priority for policymakers, academia, or social actors in general, mainly due to ethnic, religious, political, and social limitations (74).

As reported earlier, a major part of the MDGs has been at least partially accomplished. Many countries are trying to adopt a sustainable path (75). Despite generally positive outputs, global targets have not been met in some regions, particularly in sub-Saharan Africa and south Asia. Indeed, MDGs have encountered a range of common challenges (76). There are no measuring indicators regarding financial coverage/protection for health-related goals. As a result, countries have no mandatory or priority to design and implement those programs. Therefore, MDG health-related goals had focused on health service and population coverage other than financial coverage.

### Assessment of coverage

At this time, there was a good foundation for UHC. Health service coverage was extended to the basic (or limited) health service package, but not the universal health package. Indicators have been established and measured, such as immunization including more antigens, case findings, free-of-cost treatment completion of major diseases (TB, leprosy, malaria, and HIV/AIDS), four-time antenatal visit, health facility delivery, birth control, vitamin A, and iron distribution to the target population. Approximately 60–80% of the population was covered. For financial coverage, the concept of mandatory health insurance was introduced to overcome out-of-pocket and catastrophic health expenditures. Major programs were focused on mitigating child and maternal mortality in low- and middle-income countries.

### Sustainable development goal (SDG) (2015–2030): A period of financial protection in healthcare

Declaration of sustainable development goals (SDG) provides a global political commitment that can influence health financing reform (HFR) for UHC at the national level. For sustainable development, under goal 3 (ensure healthy lives and promote wellbeing for all at all ages), achieving UHC is one of the comprehensive targets for 2030. Universal health coverage is based on the principle that all people should have access to the health services they need without suffering financial hardship while accessing such services (77). This implies that an effective, efficient, and equitable health financing system is a critical and essential component that contributes to the achievement of the UHC target under the SDG declaration (78–80). It is only possible when resources are carefully managed and spent that all people could feel sustained progress toward UHC. There should be three objectives of health financing viz. equity in the use of health services, quality of care, and financial protection of progress by maintaining transparency, accountability, efficiency, and equity in resource distribution.

In SDG, both aspects of coverage (health service coverage and financial coverage) have been committed. SDG 3.8.1 has concerns about the proportion of population that can access essential quality health services. 3.8.2 is associated with mitigating household catastrophic health expenditures, with increased health insurance. Since 2015, seven years already passed, and we need to achieve the goal until 2030. Although limited financial and significant health service coverage has been achieved, there is still a great challenge to cover the universe from both perspectives by 2030.

### Assessment of coverage

In the SDGs, health services, population, and financial coverage are equally focused. The service coverage could be measured by meeting the need for family planning with modern contraception, antenatal, peripartum, and postnatal care for newborn babies, antenatal, peripartum, and postnatal care mothers to reduce maternal mortality ratio, DTP3 vaccine coverage, MCV1 coverage, LRI, and diarrhea treatment to reduce maternal and child mortality, acute lymphoid leukemia treatment, ART coverage, asthma and epilepsy treatment, appendicitis treatment, paralytic ileus and intestinal obstruction treatment, tuberculosis, diabetes and ischemic health disease (IHD) treatment, stroke, chronic kidney disease (CKD) and COPD treatment, and cervical, breast, uterine, and colon/rectum cancer treatment. For financial coverage, health insurance should be mandatory. Advanced health insurance packages would be available by co-payments so that there should be a significant reduction on OOP and catastrophic health expenditure (CHE). Likewise, total health expenditures (individual and government) would increase. Particularly, the government health expenditure (GHE) should be increased because it is not an expenditure but an investment in the people.

**Figure 1:**
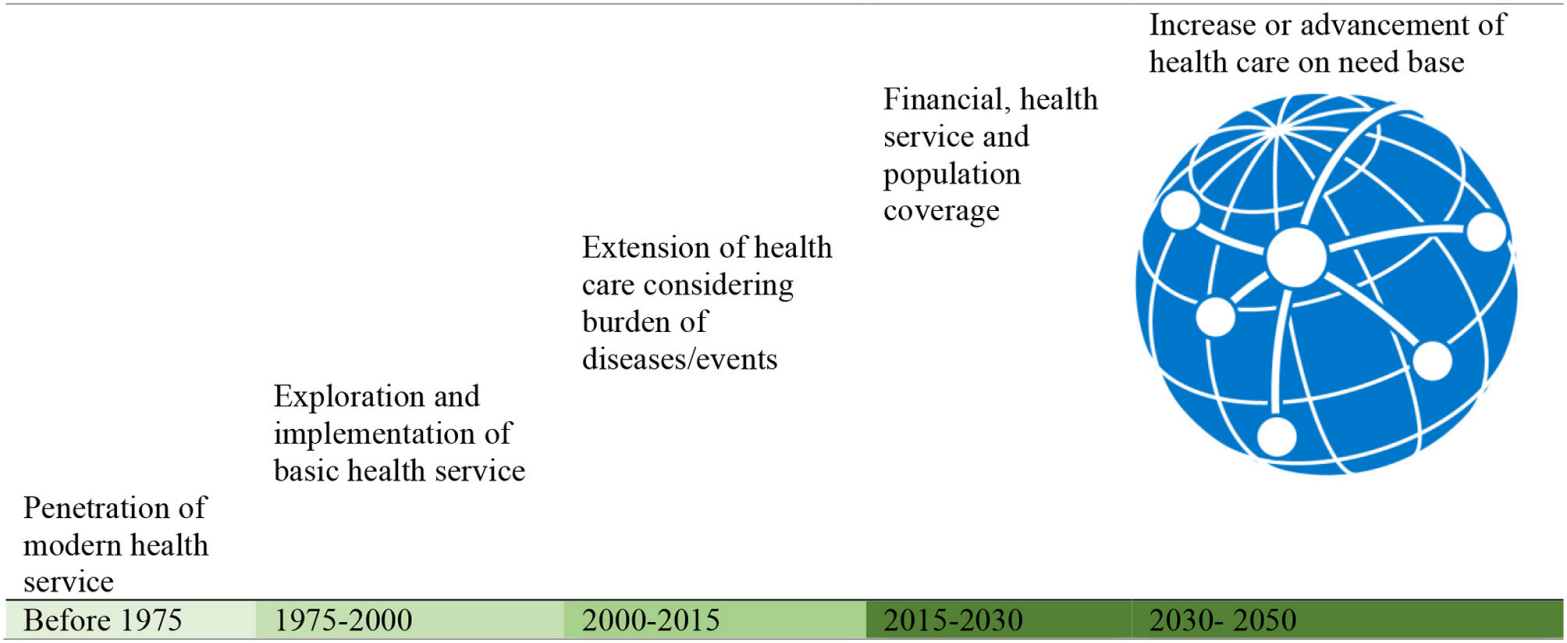
Universal health coverage (UHC) evolution, development, and destination.

The following Figure 1 shows different step-by-step evolution, development, and destination of healthcare over time. These evolution and development have been extended considering financial strength, the needs of people, chronological innovation in medical science, the use of information technologies to adopt healthcare, research in health services, and their replication in different countries.

## Future challenges and ways forward

### Healthcare coverage in public health emergencies

A public health emergency like a global pandemic is a global challenge for everyone. Public health practitioners, policymakers, researchers, scientists, and public leaders do not have a clear picture of how to handle the situation in terms of the supply chain, breaking disease transmission, economic mobility, and so on. It means that it is an extra challenge to manage financial protection for the at-risk population. In the 13th century, there was a bubonic plague that killed 200 million people in the early period of the 19th century. There was another pandemic of Spanish flu affecting approximately 100 million people. At the end of the 19th century, the global HIV pandemic started. It is still going. Its death volume was more than 35 million. More than 6 million people died recently as a result of the COVID-19 pandemic (81, 82). When universal health coverage evolved, the most important goal was financial coverage in complex situations. However, during a pandemic, it is very challenging to achieve financial protection (83). Developing UHC is the most important issue nowadays.

### Healthcare coverage in cross-country traveling and countries with an open border

After globalization and trading, many people travel internationally. Approximately 2 million people travel each day from one country to another (84). Due to long travel, business work, food habit, and weather factors, there is a high risk of getting sick. To travel across the country, full health insurance plan is needed, and authentic international organization could arrange it. There are very optional and limited plans of health insurance for travel and flight. Similarly, many people keep their regular medicine during travel. However, this regular medicine might be missed sometimes. There is no discussion of a medical plan during international traveling. It is necessary to add those plans as mandatory so that any health emergency individual could use healthcare. People living in boarder areas of two country often problem with health service access and financial assurance'. The recording and reporting of data regarding health service utilization may not be consistent, and there is always a chance of under-reporting, overlap, and verification of patients/clients. So, bilateral or international mechanisms are necessary to solve those challenges.

### Addressing the issues of migrant people

According to the UN, the estimated number of international migrants worldwide has increased in the past 20 years, between 2000 and 2020, reaching 281 million in 2020 (85). In these 20 years, the international migration trend increased by approximately 2% annually. By the end of the year, ~70.8 million individuals were forcibly displaced worldwide due to persecution, human rights violations, political conflict, war, and so on (86). According to the most recent estimates, 7.7 million people were displaced by the Russia-Ukraine War, which was equivalent to 17.5% of the entire population of Ukraine (87). Those people left their homes and everything behind in a desperate attempt to escape death and destruction. Even in legal migration, it is very hard to maintain health service access, equity, and quality. In illegal migration/displacement, there is a great challenge to survive and obtain financial coverage for health services far away. Now, it is a high time to think about how to assure basic health service for migrant people and incorporate it within the system of UHC.

### Integrating SHP with UHC

There are many resources under social security and protection. Healthcare is a neglected issue in social security programs. However, the focus program under social security is retirement token money. On the contrary, the key challenge during retirement is affordable healthcare. During the retirement period, there is limited coverage under healthcare, especially in the Medicare program due to an insufficient pooled fund. SHP could contribute to health financing for UHC. Seguro, a popular and effective program in Mexico, is a social health protection program that is effective in achieving UHC (88). There is the possibility of integration and collaboration between social health protection and UHC through information and communication technology (ICT) (89). For expedited achievement of UHC, better financial health protection with people integration or close collaboration between SHP and UHC is necessary.

### Enhancing individual responsibility for health

Health is not only the responsibility of state and individual right but also a personal responsibility. There are many circumstances where health risks can be prevented and minimized. Lifestyle choices like eating, drinking, regular checkup, workplace safety, and safe driving are individual efforts and they significantly contribute to health and establishing wellbeing (90).

### Addressing the global needs-based management of the health workforce

Human resources for health (HRH), especially those fit-for-purpose and fit-to-practice, are key to the UHC, mainly to the expansion of health service coverage and the benefits packages. A review article, which explores the policy lessons on HRH from four countries (Brazil, Ghana, Mexico, and Thailand) that have achieved sustained improvements in UHC, identifies that for effective service coverage, further attention on availability, accessibility, acceptability, and quality (AAAQ) of HRH are imperative. It also suggests partnerships involving health and non-health actors for the success of such HRH production (88). However, from the deprival aspects, the WHO's policy guiding document on global HRH for the UHC and the SDG underscores that only a 17% reduction of a total of 17.4 million deficit HRH in 2013 is projected to achieve in 2030 and still there will be a lack of around 14.5 million HRH globally, and the largest shortages will be seen in South-East Asia (6.9 million) and Africa (4.2 million). The document also focuses on the imperativeness that the developing countries should provide substantial efforts to the development, recruiting, and retention of HRH, and further recommends that the workforce that we recruit should be skilled to adopt the service delivery models emphasizing the PHC approach (91). Another HRH review paper mainly focusing on the African region emphasizes producing HRH for strategic leadership, instilling proper ethos and values, and then recruiting with equitable allocation in rural and underserved areas (92). Fundamentally, healthcare is a highly qualified service-based industry. Therefore, the production, training, mentoring, and mobilization of health human resources significantly impact the access and quality healthcare (93).

## Conclusion

Practically, UHC is an ambitious goal for every single citizen of the world, regardless of income, race, ethnicity, and geography. It is a guarantee of health services in terms of equity, access, quality, and affordability. Since the evolution of PHC, there have been tremendous achievements, mainly in health service accessibility and availability worldwide. It is a triangular balance of healthcare that considers people's needs, affordability, and innovative service models with state responsibility. Average life expectancy has increased by around 25 years. Maternal and child mortality were reduced substantially; hunger and severe malnutrition were negligible; and there were high immunization coverages for major fatal diseases, including HPV, measles, cholera, and typhoid. Still, there is a challenge in providing financial guarantees for public health emergencies, cross-country traveling, healthcare for migrant people, and the collaboration of healthcare with social protection schemes. A major portion of the health budget in the majority of countries is still allocated to tertiary and super-specialized care. Due to operational inefficiency, many countries still fail to recognize health as a right in their constitutions with ineffective implementations of policies, although they have the right policies. Country-specific policy practices such as health transformation plan (HTP) in Iran and Turkey (94), health system reform in Mexico (95), and integration of health insurance in the Republic of Korea (96) could be the best references for low- and middle-income countries. There is clear and big picture regarding UHC in this paper but there is also a clear boundary on it. So, UHC is not free health care, it is not donor funded program, the implementation strategies and practices may not consistent and it is not specific program intervention. To address challenges, a major policy departure might be essential. Moreover, a collaboration between the ILO and WHO by integrating SHP and UHC, research collaborations, and experience sharing could mitigate those challenges. Specific programs are essential in promoting individual responsibility for their health, particularly risk minimization and wellbeing, addressing the needs-based gap of HRH, strategic community participation and engagement, and utilizing ancient health systems with appropriate trade-offs of utilities.

Therefore, our study explored the different aspects of UHC, namely, historical developments, current, and future challenges. This is a hybrid type of study because the literature used in this study is research and policy-related and useful for policymakers, researchers, government agencies, and international organizations. In spite of those implications of our studies, there are some limitations too. First, we did not use specific review protocols, and we did not focus on counting the article as a review study. Similarly, the presentation of writing flow might be subjective and pragmatic rather than purely academic.

## Author contributions

CR conceptualized, designed, prepared, reviewed, and led the article. SA, CA, and C-BK thoroughly and periodically reviewed and updated the article. CA primarily addressed reviewers' comments and edited and prepared the revised version. All authors reviewed the final version of the manuscript and agreed to its submission.
